# Immunization with the H5N1 Recombinant Vaccine Candidate Induces High Protection in Chickens against Vietnamese Highly Pathogenic Avian Influenza Virus Strains

**DOI:** 10.3390/vaccines8020159

**Published:** 2020-04-02

**Authors:** Hang Thi Thu Hoang, Chi Hung Nguyen, Ngan Thi Thuy Nguyen, An Dang Pham, Hang Thi Thu Nguyen, Thanh Hoa Le, Hanh Xuan Tran, Ha Hoang Chu, Nam Trung Nguyen

**Affiliations:** 1 Institute of Biotechnology (IBT), Vietnam Academy of Science and Technology (VAST), Hanoi 100000, Vietnam; hanghtt@ibt.ac.vn (H.T.T.H.); nguyenhungchi@gmail.com (C.H.N.); nguyenthuyngan261095@gmail.com (N.T.T.N.); anphamdang92@gmail.com (A.D.P.); imibtvn@gmail.com (T.H.L.); chuhoangha@ibt.ac.vn (H.H.C.); 2 Graduate University of Science and Technology (GUST), VAST, Hanoi 100000, Vietnam; 3 Vietnam Forestry University, Xuan Mai, Chuong My, Hanoi 100000, Vietnam; thuhangvfu@gmail.com; 4 National Veterinary Joint Stock Company (NAVETCO), 29 Nguyen Dinh Chieu, Dist 1, Ho Chi Minh City 700000, Vietnam; tranxuanhanhnavetco1@gmail.com

**Keywords:** avian influenza, HPAI, recombinant strain, reverse genetics, vaccine

## Abstract

Vietnam is one of the countries most affected worldwide by the highly pathogenic avian influenza (HPAI) virus, which caused enormous economic loss and posed threats to public health. Over nearly two decades, with the antigenic changes in the diversified H5Ny viruses, the limited protective efficacy of the available vaccines was encountered. Therefore, it is necessary to approach a technology platform for the country to accelerate vaccine production that enables quick response to new influenza subtypes. This study utilized a powerful reverse genetics technique to successfully generate a recombinant H5N1 vaccine strain (designated as IBT-RG02) containing two surface proteins (haemagglutinin (HA) and neuraminidase (NA)) from the HPAI H5N1 (A/duck/Vietnam/HT2/2014(H5N1)) of the dominant clade 2.3.2.1c in Vietnam during 2012–2014. Importantly, the IBT-RG02 vaccine candidate has elicited high antibody titres in chickens (geometric mean titre (GMT) of 6.42 and 6.92, log_2_ on day 14 and day 28 p.i., respectively). To test the efficacy, immunized chickens were challenged with the circulating virulent strains. As results, there was a high protection rate of 91.6% chickens against the virulent A/DK/VN/Bacninh/NCVD-17A384/2017 of the same clade and a cross-protection of 83.3% against A/duck/TG/NAVET(3)/2013 virus of clade 1.1. Our promising results showed that we can independently master the reverse genetics technology for generation of highly immunogenic vaccine candidates, and henceforth, it is a timely manner to reformulate avian influenza virus vaccines against variable H5 clade HPAI viruses in Vietnam.

## 1. Introduction

Since the first appearance of the H5N1 epidemic in 2003, this highly pathogenic avian influenza (HPAI) virus has circulated annually in Asia under different subtypes. Vietnam is among the countries that have suffered the most serious economic losses from H5N1 worldwide, with the destruction of approximately 50 million poultry from more than 3000 outbreaks being reported between 2003 and 2019, causing a yearly loss of 0.5–1.8% of gross domestic product [1,2,3]. In addition, Vietnam is the country with the third highest number of infected (127) and fatal (64) cases in humans [4]. Due to traditional poultry-raising practices on a small scale, free trade in poultry between neighbouring countries, and direct exposure to poultry in live bird markets, there is a significantly high risk of viral transmission. Therefore, influenza management strategies are necessary to reduce detrimental effects to both humans and poultry.

To date, vaccination has been the best method for protecting poultry from influenza A infection. During the last decade, the awareness of influenza prevention in the Vietnamese farming community has considerably increased. Moreover, supplementation with avian influenza vaccines was better supported by domestic veterinary drug companies. However, influenza control programmes still have several limitations, leading to a low percentage of vaccination coverage all over the 63 provinces of Vietnam [5]. In particular, due to the frequent antigenic changes of this RNA virus, new HPAI strains may occur and thus reduce the effectiveness of available vaccines. Therefore, the generation of new seed viruses that match current influenza antigens is one of the most important issues facing vaccine producers.

So far, reverse genetics technology represents an effective approach for recombinant virus generation with many advantages, including considerably reduced production time and rational design of mutated genotypes [6,7]. The WHO has reported NIBRG-14, NIBRG-301, and IDCDC-RG12 as available vaccine candidates produced by bidirectional plasmid-based reverse genetics using haemagglutinin (HA) and neuraminidase (NA) segments from Vietnam H5N1 isolates [8,9]. All vaccine manufacturers in Vietnam use the classical production process of growing imported candidate viruses in chicken embryonated eggs, meaning that national production relies heavily on international collaborations. To master the development of domestic vaccine technology, this study aimed to utilize the well-established reverse genetics system to quickly generate new seed viruses with HA and NA antigens from the currently circulating H5N1 (clade 2.3.2.1c) in Vietnam.

## 2. Materials and Methods

### 2.1. Virus and Cell Culture

Human embryonic kidney 293T (HEK-293T) and Madin-Darby canine kidney (MDCK) cells were provided by ATCC (Manassas, VA, USA) and maintained in Dulbecco’s modified Eagle’s medium (DMEM) and minimum essential medium (MEM), respectively. Both of these media were supplemented with 10% foetal bovine serum (FBS) and 1% penicillin/streptomycin (Sigma). Two virulent H5N1 strains of clade 1.1 (A/duck/TG/NAVET(3)/2013) and clade 2.3.2.1C (A/DK/VN/Bacninh/NCVD-17A384/2017) (abbreviated as NAVET-2013 and NCVD-2017, respectively) were propagated in specific pathogen-free (SPF) eggs at the National Veterinary Joint Stock Company (NAVETCO) for further challenge experiments.

### 2.2. Plasmids

For reverse genetics techniques, the pHW2000 vector containing the dual promoter/terminator system was generously provided by the Department of Infectious Diseases, St. Jude Children’s Research Hospital (Memphis, TN, USA). The multi-basic cleavage site of the wild-type HA gene from the A/Vietnam/HT02/2014 H5N1 (clade 2.3.2.1c) isolate [10] was replaced with the motif of a monobasic region (Q*RERRRKR* ↓ GL → Q*RETR* ↓ GL), as previously described [11,12]. HA and NA artificial genes were synthesized by PHUSA Biochem Ltd. Co. (Cantho, Vietnam) and cloned into pHW2000. Each of six other backbone segments (PB1, PB2, PA, NP, M, and NS) from the PR8 H1N1 strain was also inserted into pHW2000. All eight plasmids were sequenced before transfection experiments.

### 2.3. Rescue of Recombinant Virus

The H5N1 recombinant virus was generated by reverse genetics using the transfection protocol as previously described [6]. Briefly, a mixture of all eight plasmids in Opti-MEM I was preincubated with Lipofectamine™ 2000 (Invitrogen, Carlsbad, CA, USA) at room temperature for 20 min, and then, this inoculant was added to 293T cells in six-well plates that were pre-seeded overnight at a density of 0.8–1 × 10^6^ cells per well. After 6 h, the DNA-transfection mixture was replaced by Opti-MEM I, and the cells were further incubated at 37 °C and 5% CO_2_. After 24 h, Opti-MEM I containing N-tosyl-L-phenylalanine chloromethyl ketone (TPCK)-treated trypsin was added to the cells to a final concentration of trypsin of 1.0 µg/mL. The cell supernatant at 48 h after transfection was collected and inoculated in 9–11-day-old SPF embryonated chicken eggs for further propagation of virus. After 72 h, the eggs were chilled for 1 day, and then, the allantoic fluid was harvested, serially passaged, and stored at −80 °C. The 50% embryo infectious dose (EID_50_) was determined by the Reed–Muench method [13]. The presence of recombinant virus was confirmed by two-step RT-PCR using Hoffmann universal primers [14] and by hemagglutination assay (HA) with 0.5% suspension of chicken red blood cells. Titration of virus at each passage was performed by standard MDCK plaque assay. The mutations of HA and the genetic stability of recombinant virus were carefully checked by sequencing. The pathogenicity of the rescued virus was evaluated by the intravenous pathogenicity index (IVPI) following the standard procedure described in the Terrestrial Manual of the World Organisation for Animal Health (OIE) [15]. The recombinant virus was inactivated by 0.12% formalin for 24 h and emulsified in NAVETCO adjuvant (at a 1:1 ratio) for experimental vaccine preparation.

### 2.4. Chicken Immunization and Antibody Titration

A total of thirty-four 21-day-old SPF chickens were divided into three groups. For the experimental vaccinated group (*n* = 12), each chicken was intramuscularly injected with 0.2 mL of IBT-RG02 containing 64 HA units (HAU). Phosphate-buffered saline (PBS) and NAVET-Vifluvac commercial vaccine (NIBRG-14 WHO candidate strain) were used for the negative (*n* = 10) and positive (*n* = 12) control groups, respectively. Sera were collected at day 14 and day 28 postvaccination. A haemagglutination inhibition (HI) assay was applied for antibody titration using the homologous and heterologous antigens (clade 2.3.2.1c and clade 1.1, respectively). Geometric mean titre (GMT) represents the mean antibody titre of each group. Sera with GMT > 2 (log_2_) were considered as positive.

### 2.5. Challenge Study in Chickens

At day 28 post-vaccination, the immunized and control chickens were challenged with NAVET-2013 and NCVD-2017 HPAI isolates in separate units of biosafety cabinets. The challenge dose for each virus was 10^6.5^ ELD_50_ per chicken through the intraocular route. Chickens were observed and recorded daily for morbidity and mortality during a 21-day period post-challenge. The sample collection and challenging procedures were approved by the Department of Animal Health (DAH) of the Vietnamese Ministry of Agriculture and Rural Development (MARD). All laboratory works for implementation of the project were performed in accordance with the “3Rs” and the relevant rules of both Institute of Biotechnology (IBT, Vietnam) and NAVETCO ethics committees under the decision number 1963/QD-BKHCN.

### 2.6. Data Analysis

Statistical analysis between different experimental groups was performed by the Mann–Whitney U-test with SPSS software (SPSS, Chicago, IL). A *p*-value smaller than 0.05 was considered significant. All sequencing analyses were performed by the BioEdit software (version 7.2.6.1, Raleigh, NC, USA) and the Basic Local Alignment Search Tool (BLAST) from the NCBI website.

## 3. Results

### 3.1. Generation of the Recombinant H5N1 Virus (Clade 2.3.2.1c)

In this study, we attempted to construct a recombinant virus containing two glycoprotein surface antigens (HA and NA) from a circulating influenza A strain in Vietnam on a PR8 virus background. To this end, A/duck/Vietnam/HT2/2014(H5N1) (abbreviated as HT2-2014) was selected due to its high prevalence as an HPAI variant of the 2.3.2.1c clade that has spread widely in multiple avian species (e.g., duck, chicken, gull, and quail) from northern to southern provinces since 2010 [10,16]. This strain was phylogenetically classified separately from Chinese, Mongolian, Laotian, and Indonesian strains of the same clade [10,17]. Our RT-PCR results from three independent transformation experiments showed that the 293T cell system was efficient to rescue the HA-NA(HT2)-PR8 virus (namely, IBT-RG02) entirely from the eight pHW2000 plasmids by reverse genetics. Importantly, this recombinant strain (passage 0–P0) can directly grow at high yields in embryonated chicken eggs without an MDCK-infected intermediate step. After only one passage in eggs, the collected allantoic fluid from individual samples (passage 1–P1) reached an average viral titre of 8.30 PFU/mL (log_10_) in the MDCK plaque assay and HA titres of 9.37 (log_2_) in the haemagglutination assay (Table 1). Sequencing data from all eight gene segments of IBT-RG02 showed that this strain had 100% genetic identity compared to the original DNA inserts and that the multi-basic region in the HA cleavage site was converted into monobasic motif as desired.

To further test the capacity for pathogenicity switching in the recombinant strain in vivo, additional propagation of the P1 virus was performed in eggs, and samples were carefully checked at each passage. At the fifth passage (P5), all samples retained an EID_50_ of 8.30/mL (log_10_), an HA titre of 9.80 (log_2_), and viral titre of 8.86 PFU/mL (log_10_) (Table 1), showing only small variations between independent transfections. In addition, no nucleotide mutation was found, indicating the high stability of our seed virus for subsequent experiments. Next, the attenuation of our IBT-RG02 seed virus was examined by intravenous injection of 10^6.3^ EID_50_/0.1 mL into each of ten 3-week-old chickens. Similar to the PBS-injected controls, all IBT-RG02-injected chickens survived after 14 days without any clinical signs (Table S1), confirming that the high pathogenicity of the HT2-2014 strain was successfully abolished [15].

### 3.2. Immunogenicity Characterization of the IBT-RG02 Strain in Chickens

To determine the immunogenicity of the recombinant strain, an HI test was performed for sera from chickens injected with 0.2 mL of formalin-inactivated vaccine containing 64 HAU of IBT-RG02 seed virus. Our results showed that vaccination could essentially elicit an antibody response in 100% of IBT-RG02-innoculated chickens (Table 2). The mean GMT HI titres against the homologous antigens (IBT-RG02) were 6.42 and 6.92 (log_2_) at day 14 and day 28 p.i., respectively, compared to the absence of anti-H5 antibody in PBS controls (GMT < 1/2 (log_2_)). Moreover, we observed the cross-reactivity of these serum antibodies against the heterologous HA antigens from the clade 1.1 recombinant IBT-RG01 virus (see Discussion for more details) but with approximately two logs lower than that of the homologous antigens.

### 3.3. Protection Efficacy of IBT-RG02 in Chickens

The efficacy of our recombinant seed virus was demonstrated by challenging IBT-RG02-vaccinated chickens with the HPAI H5N1 strain of the same clade 2.3.2.1c (NCVD-2017). At day 21 after challenge, 91.6% of chickens survived and remained healthy, whereas all non-vaccinated chickens died quickly in three days in contrast to the 100 % protection provided by the NAVET-Vifluvac commercial vaccine. To further evaluate the potency of the seed virus, vaccinated chickens were challenged with the H5N1 strain of clade 1.1 possessing the heterologous HA antigen (NAVET-2013). Protection was observed but with a slightly lower percentage (83.3%) compared to 91.6% protection by NAVET-Vifluvac (Table 3). These results showed that IBT-RG02 has a strong ability to protect chicken from H5N1 of at least the two clades tested in the study and thus could potentially be used as a local avian vaccine candidate.

## 4. Discussion

Due to the frequent evolutionary characteristics of influenza A virus, tradition of small-scale farming, and high percentage of waterfowl species in total poultry number in Vietnam, this country continuously appears in global influenza maps and is considered a high-risk environment for potentially pandemic strains, such as H5N1 [18]. The occurrence of new H5N1 strains, such as viruses of clade 2.3.2.1 and 2.3.4.4 in Vietnam since 2013, has continued to present difficulties to the country, despite strengthening disease control strategies at both the national and local government levels. Vietnam has received multiple lessons from a significant reduction in the efficacy of available vaccines. Thus, it is crucial to update vaccine strains to accommodate new emerging viruses.

This study successfully applied reverse genetics to generate a recombinant H5N1 virus strain with antigens originating from an HPAI Vietnamese isolate. It is important to note that HT2-2014 belonged to clade 2.3.2.1c, which was dominant in many provinces of Vietnam during the period of 2010–2015 when our study began. At that time, the failure of Re-1(clade 0)/Re-5(clade 2.3.4) Harbin imported vaccines in challenging clade 2.3.2.1a, b, c wild-type strains led the Vietnamese government to announce the pending release of these vaccines, to import the Re-6 vaccine (clade 2.3.2), and to call for intensive research in influenza vaccine development [10]. Therefore, one of the main achievements of this study is to establish a reverse-genetics platform in Vietnam for rapid response to novel variants of influenza, especially in the event of a future pandemic.

Our first step was to modify the HA gene in order to reduce the pathogenicity of the HPAI virus, one of the most important safety issues in influenza vaccine production. In this study, at least up to the fifth passage of the IBT-RG02 virus, all deduced amino acid sequences of HA showed that three arginines (R) (highlighted in bold) at the multi-basic cleavage site (Q*R*E**RRR**K*R*G, position 338–346) were completely removed and that one lysine (K) residue (underlined) at position 344 was replaced by threonine (T) as desired. In addition, to prevent pathogenicity reversion, three nucleotides coding for arginines (italics) at two ends of the pathogenic region were modified from AGA to CGA (Figure S1). Moreover, the theoretical modification from an HPAI to an LPAI strain was also confirmed in vivo by the IVPI in IBT-RG02 inoculated chickens. Besides, based on the data of HA and viral titre, we concluded that the recombinant IBT-RG02 strain was successfully created from the original HT2-2014 H5N1 strain by reverse genetics within 10–12 weeks (including HA and NA construct design and artificial synthesis, cell transformation, and virus propagation).

In our study, 64 HAU of the IBT-RG02 strain was able to elicit mean HI titres between 6.42 and 6.92 (log_2_) in chickens after immunization, which are similar to those of H5N1 recombinant strains from other reports [19,20,21]. However, these studies have differences in doses and schedules of vaccination. For example, Kandeil [20] immunized one-week-old chickens with 256 HAU of recombinant H5N1 virus. On the other hand, under a higher dose of immunization (640–3200 HAU), the recombinant Miyazaki H5N1 strain (clade 2.2) induced a higher HI titre (12.31–23.78, log_2_) [22] but could not reach 100% of positive sera, as in our study (12/12, HI ≥ 4 log_2_). Besides, the cross reactivity of the antibody against the heterologous HA (clade 2.3.2.1c, Akita strain) was observed in only one of eight vaccinated chickens with substantially lower HI titre (5.45, log_2_). In summary, the IBT-RG02 strain is a potential antigen candidate according to WHO guidelines for the production of influenza vaccine [8].

In principal, the “6 + 2” reverse genetics allows the generation of a virus strain from a plasmid set containing all eight segments of the influenza A genome [6]. However, it has been demonstrated that some recombinant strains did not grow in eggs to quantities sufficient for immunization tests and further vaccine production; therefore, these strains could not be used as vaccine candidate strains [20,22,23]. In our study, a recombinant strain accommodating HA and NA from ST-2009 HPAI H5N1 (clade 1.1) was also produced on the PR8 background but was unable to replicate in eggs (negative in the HA assay). When the NA gene of ST-2009 was replaced by the NA gene of HT2-2014, a significant improvement in egg infection was observed (data not shown), indicating the importance of both NA itself and the interaction between surface H5N1 antigens and other internal PR8 proteins. However, the HI titre of this ST-2009-H5/HT-2014-N1/PR8 strain (namely IBT-RG01) in chickens after immunization was lower than that of the IBT-RG02 strain (5.50 and 6.17 (log_2_) at day 14 and day 28 p.i., respectively). Moreover, this IBT-RG01 strain exhibits low protection (58.3%) against homologous challenge in contrast to the high protection (91.6%) of IBT-RG02, emphasizing the importance of individual gene constellations in influenza reverse genetics. These results are in keeping with previous observations that HA is a highly important antigen in inducing antibody production to protect chickens after influenza infection.

In conclusion, our successful initial results help to establish a reliable platform for national rapid response to new variants of influenza A virus according to yearly WHO recommendations. Although currently circulating strains belong to new H5N6 strains evolving from the 2.3.4.4 clade, important amino acid residues are conserved between HA proteins of the 2.3.2.1c and 2.3.4.4 clades, including the receptor-binding site, cleavage site (RERRRKR), glycosylation sites, and antigenic sites. Interestingly, Zhang et al. (2019) recently reported that a novel reassortant strain, A/*Streptopelia decaocto*/Jiangxi/G6/2016 (H5N6), was found to have the HA gene belonging to clade 2.3.2.1c [24]. It is indicated that nucleotide sequences of most preexisting H5N1 influenza viruses of clades 1.1.1/1.1.2, 2.3.2.1a/b/c, and 2.3.4 and the current H5N6 circulating in Vietnam share a high similarity with isolates from southern China [10,25]. Therefore, it is possible that new H5N6 viruses with 2.3.2.1c-original HA (similar to the Jiangxi strain) will be introduced to Vietnam and that reassortment activities may occur. Therefore, in addition to the protection efficacy of IBT-RG02 against our recent Vietnamese H5N1 isolate (NCVD-2017), challenge tests of immunized chickens against new H5N6 Vietnamese isolates are underway to further evaluate the cross-protection efficacy of this candidate vaccine virus.

## Figures and Tables

**Table 1:** Analysis of the recombinant H5N1 virus at different passages in specific pathogen-free (SPF) eggs.

Passage	HA Titer (log_2_)	Viral Titer (PFU/mL, log_10_)	EID_50_/mL (log_10_)	Sequence Identity ^d^ (%)
P1 ^a^	9.37	8.30	8.00	100
P3 ^b^	9.80	ND ^c^	ND	100
P5	9.80	8.86	8.30	100

^a^ Data for P1 represent the average value of all allantoic samples from three independent transfection and egg infection experiments; ^b^ Data for P3 and P5 represent the average value of ten allantoic samples from inoculated eggs; ^c^ ND: not determined; ^d^ Nucleotide sequence identity of IBT-RG02 backbone segments (PB1, PB2, PA, NP, M, and NS) and haemagglutinin (HA) and neuraminidase (NA) genes compared to the respective inserts of the pHW2000 plasmids.

**Table 2:** Antibody production in chickens after immunization with the rescued recombinant H5N1 virus.

Immunization ^a^	Antigen					
	Clade 2.3.2.1c ^b^			Clade 1.1 ^c^		
	Day 0 b.i. ^d^	Day 14 p.i. ^e^	Day 28 p.i.	Day 0 b.i.	Day 14 p.i.	Day 28 p.i.
Control (PBS)	<1/2 ^f^	<1/2	<1/2	<1/2	<1/2	<1/2
IBT-RG02	<1/2	6.42 (12/12) ^g^	6.92 (12/12)	<1/2	4.50	4.92
NAVET-Vifluvac ^h^	<1/2	5.75 (12/12)	6.33 (12/12)	<1/2	5.25	7.08

^a^ Vaccines were given to 3-week-old chickens via the intramuscular (IM) route as a single dose; ^b^ Clade 2.3.2.1c: the recombinant IBT-RG02 viruses were used as homologous antigens in HI test; ^c^Clade 1.1: the recombinant IBT-RG01 viruses were used as heterologous antigens in HI test; ^d^b.i.: before immunization; ^e^ p.i.: post immunization; ^f^ The haemagglutination inhibition (HI) titers were represented as geometric mean titre in log_2_ scale; ^g^ Number of positive sera/total; ^h^ The NAVET-Vifluvac vaccine was used as positive control.

**Table 3:** Protection of 3-week-old chickens against HPAI challenge after immunization with IBT-RG02.

Immunization ^a^	Challenge strains^b^					
	NAVET-2017			NCVD-2013		
	Total Chicken Number	Survival Number	Protection Rate (%)	Total Chicken Number	Survival Number	Protection Rate (%)
**Control (PBS)**	05	0/5	0.0	05	0/5	0.0
**NAVET-Vifluvac ^c^**	12	12/12	100	12	11/12	91.6
**IBT-RG02**	12	11/12	91.6	12	10/12	83.3

^a^ Vaccines were given to 3-week-old chickens via the intramuscular (IM) route as a single dose. ^b^ Challenging viruses were given to the vaccinated chickens via the intraocular (IO) route. ^c^ The NAVET-Vifluvac vaccine was used as positive control.

## Supplementary Materials

The following are available online at https://www.mdpi.com/2076-393X/8/2/159/s1, Figure S1. Stability of the artificial HA gene from the rescued virus was validated by sequencing. Table S1. Safety analysis of the rescued recombinant H5N1 virus in immunized chickens.
