# Supplementary material for: Immunization with the H5N1 Recombinant Vaccine Candidate Induces High Protection in Chickens against Vietnamese Highly Pathogenic Avian Influenza Virus Strains
Source: Vaccines (Basel). 2020 Apr 2;8(2):159. doi: 10.3390/vaccines8020159 (PMC7348806; doi:10.3390/vaccines8020159)
Supplement: Supplementary file 1 [file vaccines-08-00159-s001.pdf]

vaccines

Supplementary Material

```
                         10        20        30        40        50        60        70        80
                 ....|....|....|....|....|....|....|....|....|....|....|....|....|....|....|....|
HT2_2014_origin  ------------------------------------------------------ATGGAGAAAATAGTTCTTCTCTTTGC
                                                                        M  E  K  I  V  L  L  F  A
HT2_2014_muta    TAGTACTAAGCATATTCGTCTCAGGGAGCAAAAGCAGGGGTCTAATCTGTCAAA..........................
                                                                        M  E  K  I  V  L  L  F  A

                         90       100       110       120       130       140       150       160
                 ....|....|....|....|....|....|....|....|....|....|....|....|....|....|....|....|
HT2_2014_origin  AACAATCAGCCTTGTCAAAAGCGATCATATTTGCATTGGTTATCATGCAAATAACTCGACAGAGCAGGTTGACACAATAA
                   T  I  S  L  V  K  S  D  H  I  C  I  G  Y  H  A  N  N  S  T  E  Q  V  D  T  I
HT2_2014_muta    ................................................................................
                   T  I  S  L  V  K  S  D  H  I  C  I  G  Y  H  A  N  N  S  T  E  Q  V  D  T  I

                        170       180       190       200       210       220       230       240
                 ....|....|....|....|....|....|....|....|....|....|....|....|....|....|....|....|
HT2_2014_origin  TGGAAAAGAACGTTACTGTCACACATGCCCAAGACATACTGGAAAAGACACACAATGGGAAGCTCTGCGATCTAAATGGA
                 M  E  K  N  V  T  V  T  H  A  Q  D  I  L  E  K  T  H  N  G  K  L  C  D  L  N  G
HT2_2014_muta    ................................................................................
                 M  E  K  N  V  T  V  T  H  A  Q  D  I  L  E  K  T  H  N  G  K  L  C  D  L  N  G

                        250       260       270       280       290       300       310       320
                 ....|....|....|....|....|....|....|....|....|....|....|....|....|....|....|....|
HT2_2014_origin  GTGAAGCCTCTGATTTTAAAAGATTGTAGTGTAGCAGGATGGCTCCTCGGAAATCCATTGTGTGACGAATTCATCAATGT
                  V  K  P  L  I  L  K  D  C  S  V  A  G  W  L  L  G  N  P  L  C  D  E  F  I  N  V
HT2_2014_muta    ................................................................................
                  V  K  P  L  I  L  K  D  C  S  V  A  G  W  L  L  G  N  P  L  C  D  E  F  I  N  V

                        330       340       350       360       370       380       390       400
                 ....|....|....|....|....|....|....|....|....|....|....|....|....|....|....|....|
HT2_2014_origin  GCCAGAATGGTCTTACATAGTAGAGAAGCCCAATCCAGCCAATGACCTCTGTTACCCAGGAAATTTCAACGATTATGAAG
                   P  E  W  S  Y  I  V  E  K  P  N  P  A  N  D  L  C  Y  P  G  N  F  N  D  Y  E
HT2_2014_muta    ................................................................................
                   P  E  W  S  Y  I  V  E  K  P  N  P  A  N  D  L  C  Y  P  G  N  F  N  D  Y  E

                        410       420       430       440       450       460       470       480
                 ....|....|....|....|....|....|....|....|....|....|....|....|....|....|....|....|
HT2_2014_origin  AATTGAAACACCTATTGAGCAGGATAAACCATTTTGAGAAAATACAGATCATCCCCAAAGATTCTTGGTCAAACCATGAA
                 E  L  K  H  L  L  S  R  I  N  H  F  E  K  I  Q  I  I  P  K  D  S  W  S  N  H  E
HT2_2014_muta    ................................................................................
                 E  L  K  H  L  L  S  R  I  N  H  F  E  K  I  Q  I  I  P  K  D  S  W  S  N  H  E

                        490       500       510       520       530       540       550       560
                 ....|....|....|....|....|....|....|....|....|....|....|....|....|....|....|....|
HT2_2014_origin  GCCTCATTGGGGGTGAGCGCAGCATGTTCATACCAGGGAAATTCCTCCTTCTTCAGAAATGTGGTGTGGCTTATCAAAAA
                  A  S  L  G  V  S  A  A  C  S  Y  Q  G  N  S  S  F  F  R  N  V  V  W  L  I  K  K
HT2_2014_muta    ................................................................................
                  A  S  L  G  V  S  A  A  C  S  Y  Q  G  N  S  S  F  F  R  N  V  V  W  L  I  K  K

                        570       580       590       600       610       620       630       640
                 ....|....|....|....|....|....|....|....|....|....|....|....|....|....|....|....|
HT2_2014_origin  GGACAATGCATACCCAACAATAAAGAAAGGCTACAATAATACCAACCGAGAAGATCTATTGATACTGTGGGGAATCCACC
                   D  N  A  Y  P  T  I  K  K  G  Y  N  N  T  N  R  E  D  L  L  I  L  W  G  I  H
HT2_2014_muta    ................................................................................
                   D  N  A  Y  P  T  I  K  K  G  Y  N  N  T  N  R  E  D  L  L  I  L  W  G  I  H

                        650       660       670       680       690       700       710       720
                 ....|....|....|....|....|....|....|....|....|....|....|....|....|....|....|....|
HT2_2014_origin  ATCCTAATGATGAGGCGGAACAGACAAGGCTCTACCAAAACCCAACTACCTATATTTCCATTGGGACTTCAACACTAAAC
                 H  P  N  D  E  A  E  Q  T  R  L  Y  Q  N  P  T  T  Y  I  S  I  G  T  S  T  L  N
HT2_2014_muta    ................................................................................
                 H  P  N  D  E  A  E  Q  T  R  L  Y  Q  N  P  T  T  Y  I  S  I  G  T  S  T  L  N

                        730       740       750       760       770       780       790       800
                 ....|....|....|....|....|....|....|....|....|....|....|....|....|....|....|....|
HT2_2014_origin  CAGAGATTGGTACCAAAAATAGCCACTAGATCCAAAATAAACGGGCAAAGTGGCAGGATAGATTTCTTCTGGACAATTTT
                  Q  R  L  V  P  K  I  A  T  R  S  K  I  N  G  Q  S  G  R  I  D  F  F  W  T  I  L
HT2_2014_muta    ................................................................................
                  Q  R  L  V  P  K  I  A  T  R  S  K  I  N  G  Q  S  G  R  I  D  F  F  W  T  I  L

                        810       820       830       840       850       860       870       880
                 ....|....|....|....|....|....|....|....|....|....|....|....|....|....|....|....|
HT2_2014_origin  AAAACCGAATGACGCAATCCACTTCGAGAGTAATGGGAATTTCATTGCTCCAGAATATGCGTACAAAATTGTCAAGAAGG
                   K  P  N  D  A  I  H  F  E  S  N  G  N  F  I  A  P  E  Y  A  Y  K  I  V  K  K
HT2_2014_muta    ................................................................................
                   K  P  N  D  A  I  H  F  E  S  N  G  N  F  I  A  P  E  Y  A  Y  K  I  V  K  K

                        890       900       910       920       930       940       950       960
                 ....|....|....|....|....|....|....|....|....|....|....|....|....|....|....|....|
HT2_2014_origin  GAGACTCCACAATCATGAGAAGTGAAGTGGAATATGGTAACTGCAACACCAGGTGTCAGACTCCAATAGGGGCGATAAAC
                 G  D  S  T  I  M  R  S  E  V  E  Y  G  N  C  N  T  R  C  Q  T  P  I  G  A  I  N
HT2_2014_muta    ................................................................................
                 G  D  S  T  I  M  R  S  E  V  E  Y  G  N  C  N  T  R  C  Q  T  P  I  G  A  I  N
```

```
                          970       980       990       1000      1010      1020      1030
1040
                     ....|....|....|....|....|....|....|....|....|....|....|....|....|....|
HT2_2014_origin      TCTAGTATGCCATTCCACAACATACACCCTCTCACCATCGGAGAATGTCCCAAATATGTGAAATCAAACAAATTAGTCCT
                      S  S  M  P  F  H  N  I  H  P  L  T  I  G  E  C  P  K  Y  V  K  S  N  K  L  V  L
HT2_2014_muta        ......................................................................
                      S  S  M  P  F  H  N  I  H  P  L  T  I  G  E  C  P  K  Y  V  K  S  N  K  L  V  L

                          1050      1060      1070      1080      1090      1100      1110
1120
                     ....|....|....|....|....|....|....|....|....|....|....|....|....|....|
HT2_2014_origin      TGCAACTGGGCTCAGAAATAGTCCTCAAAGAGAGAGAAGAAGAAAAAGAGGACTGTTTGGAGCTATAGCAGGTTTTATAG
                       A  T  G  L  R  N  S  P  Q  R  E  R  R  R  K  R  G  L  F  G  A  I  A  G  F  I
HT2_2014_muta        .........................C....--------.CGC...........................
                       A  T  G  L  R  N  S  P  Q  R  E              T  R  G  L  F  G  A  I  A  G  F  I

                          1130      1140      1150      1160      1170      1180      1190
1200
                     ....|....|....|....|....|....|....|....|....|....|....|....|....|....|
HT2_2014_origin      AGGGAGGATGGCAGGGAATGGTAGATGGTTGGTATGGGTACCACCACAGCAATGAACAGGGGAGTGGTTACGCTGCAGAC
                     E  G  G  W  Q  G  M  V  D  G  W  Y  G  Y  H  H  S  N  E  Q  G  S  G  Y  A  A  D
HT2_2014_muta        ......................................................................
                     E  G  G  W  Q  G  M  V  D  G  W  Y  G  Y  H  H  S  N  E  Q  G  S  G  Y  A  A  D

                          1210      1220      1230      1240      1250      1260      1270
1280
                     ....|....|....|....|....|....|....|....|....|....|....|....|....|....|
HT2_2014_origin      AAAGAATCTACTCAAAAGGCGATAGACGGGGTCACCAATAAGGTCAATTCGATCATTGACAAAATGAACACTCAGTTTGA
                      K  E  S  T  Q  K  A  I  D  G  V  T  N  K  V  N  S  I  I  D  K  M  N  T  Q  F  E
HT2_2014_muta        ......................................................................
                      K  E  S  T  Q  K  A  I  D  G  V  T  N  K  V  N  S  I  I  D  K  M  N  T  Q  F  E

                          1290      1300      1310      1320      1330      1340      1350
1360
                     ....|....|....|....|....|....|....|....|....|....|....|....|....|....|
HT2_2014_origin      GGCTGTAGGAAGGGAATTTAATAATTTAGAGAGGAGAATAGAAAATTTAAACAAGAAGATGGAAGACGGATTCCTAGATG
                       A  V  G  R  E  F  N  N  L  E  R  R  I  E  N  L  N  K  K  M  E  D  G  F  L  D
HT2_2014_muta        ......................................................................
                       A  V  G  R  E  F  N  N  L  E  R  R  I  E  N  L  N  K  K  M  E  D  G  F  L  D

                          1370      1380      1390      1400      1410      1420      1430
1440
                     ....|....|....|....|....|....|....|....|....|....|....|....|....|....|
HT2_2014_origin      TCTGGACTTATAATGCTGAACTTCTGGTTCTCATGGAGAATGAGAGAACTCTAGACTTCCATGACTCAAATGTCAAGAAC
                     V  W  T  Y  N  A  E  L  L  V  L  M  E  N  E  R  T  L  D  F  H  D  S  N  V  K  N
HT2_2014_muta        ......................................................................
                     V  W  T  Y  N  A  E  L  L  V  L  M  E  N  E  R  T  L  D  F  H  D  S  N  V  K  N

                          1450      1460      1470      1480      1490      1500      1510
1520
                     ....|....|....|....|....|....|....|....|....|....|....|....|....|....|
HT2_2014_origin      CTTTACGATAAGGTCCGACTACAGCTTAAGGATAATGCAAAAGAGCTGGGAAACGGTTGTTTCGAGTTCTATCACAAATG
                      L  Y  D  K  V  R  L  Q  L  K  D  N  A  K  E  L  G  N  G  C  F  E  F  Y  H  K  C
HT2_2014_muta        ......................................................................
                      L  Y  D  K  V  R  L  Q  L  K  D  N  A  K  E  L  G  N  G  C  F  E  F  Y  H  K  C

                          1530      1540      1550      1560      1570      1580      1590
1600
                     ....|....|....|....|....|....|....|....|....|....|....|....|....|....|
HT2_2014_origin      TAATAATGAATGTATGGAAAGTGTAAGAAACGGGACGTATGACTACCCGCAGTATTCAGAAGAAGCAAGATTAAAAAGAG
                       N  N  E  C  M  E  S  V  R  N  G  T  Y  D  Y  P  Q  Y  S  E  E  A  R  L  K  R
HT2_2014_muta        ......................................................................
                       N  N  E  C  M  E  S  V  R  N  G  T  Y  D  Y  P  Q  Y  S  E  E  A  R  L  K  R

                          1610      1620      1630      1640      1650      1660      1670
1680
                     ....|....|....|....|....|....|....|....|....|....|....|....|....|....|
HT2_2014_origin      AGGAAATAAGTGGAGTAAAACTGGAATCAATAGGAATCTACCAAATACTGTCAATTTATTCAACAGTGGCGAGTTCCCTA
                     E  E  I  S  G  V  K  L  E  S  I  G  I  Y  Q  I  L  S  I  Y  S  T  V  A  S  S  L
HT2_2014_muta        ......................................................................
                     E  E  I  S  G  V  K  L  E  S  I  G  I  Y  Q  I  L  S  I  Y  S  T  V  A  S  S  L

                          1690      1700      1710      1720      1730      1740      1750
1760
                     ....|....|....|....|....|....|....|....|....|....|....|....|....|....|
HT2_2014_origin      GCGCTGGCAATCATGATGGCTGGTCTATCTTTATGGATGTGTTCCAACGGATCGTTACAGTGCAGGATCTGCATCTAA--
                      A  L  A  I  M  M  A  G  L  S  L  W  M  C  S  N  G  S  L  Q  C  R  I  C  I  *
HT2_2014_muta        ....................................................................AT
                      A  L  A  I  M  M  A  G  L  S  L  W  M  C  S  N  G  S  L  Q  C  R  I  C  I  *

                          1770      1780      1790      1800      1810      1820      1830
                     ....|....|....|....|....|....|....|....|....|....|....|....|....|....|.
HT2_2014_origin      -----------------------------------------------------------------------

HT2_2014_muta        TTGTGAATTCAGATTGTAGTTAAAAACACCCTTGTTTCTACTAATACGAGACGATATTAGTACTAAGCATA
```

Figure 1. Stability of the artificial HA gene from the rescued virus was validated by sequencing. The 5′ and 3′ noncoding sequence in HT2_2014_muta were designed according to the molecular method described in Reference [14], including conserved regions between different influenza A subtypes (underlined) and the *BsmBI* recognition site (italic). The pathogenic region of HA from the original HPAI strain (HT2_2014_origin, IBT data collection) was modified as expected (HT2_2014_muta). The red arrow indicates the removal of the pathogenic region.

Table 1. Safety analysis of the rescued recombinant H5N1 virus in immunized chickens.

Immunization[a]	Total chicken number	Survival number	Chicken number with clinical outcomes[b]	Overall IVPI[c]
**Control (PBS)**	10	10/10	0/10	0
**IBT-RG02**	10	10/10	0/10	0
**NAVET-Vifluvac[d]**	10	10/10	0/10	0

[a]Vaccines were given to 3-week-old chickens via the intramuscular (IM) route as a single dose; [b]Clinical outcomes: chickens with symptoms of flu disease after immunization (according to body weight loss and OIE manual on assessment of sick and severely sick chickens); [c]The intravenous pathogenicity index (IVPI) was scored for each chicken and the mean value was calculated; [d]The NAVET-Vifluvac vaccine was used as positive control.
